# The Anticipatory and Task-Driven Nature of Visual Perception

**DOI:** 10.1093/cercor/bhab163

**Published:** 2021-09-07

**Authors:** Sebo Uithol, Katherine L Bryant, Ivan Toni, Rogier B Mars

**Affiliations:** Donders Institute for Brain, Cognition and Behaviour, Radboud University Nijmegen, 6525 HR, Nijmegen, the Netherlands; Cognitive Psychology Unit, Institute of Psychology, Leiden University, 2333 AK Leiden, the Netherlands; Donders Institute for Brain, Cognition and Behaviour, Radboud University Nijmegen, 6525 HR, Nijmegen, the Netherlands; Wellcome Centre for Integrative Neuroimaging, Centre for Functional MRI of the Brain (FMRIB), Nuffield Department of Clinical Neurosciences, John Radcliffe Hospital, University of Oxford, Oxford OX3 9DU, UK; Donders Institute for Brain, Cognition and Behaviour, Radboud University Nijmegen, 6525 HR, Nijmegen, the Netherlands; Donders Institute for Brain, Cognition and Behaviour, Radboud University Nijmegen, 6525 HR, Nijmegen, the Netherlands; Wellcome Centre for Integrative Neuroimaging, Centre for Functional MRI of the Brain (FMRIB), Nuffield Department of Clinical Neurosciences, John Radcliffe Hospital, University of Oxford, Oxford OX3 9DU, UK

**Keywords:** visual Categorization, MVPA, FMRI, conceptual knowledge

## Abstract

Humans have a remarkable capacity to arrange and rearrange perceptual input according to different categorizations. This begs the question whether the categorization is exclusively a higher visual or amodal process, or whether categorization processes influence early visual areas as well. To investigate this we scanned healthy participants in a magnetic resonance imaging scanner during a conceptual decision task in which participants had to answer questions about upcoming images of animals. Early visual cortices (V1 and V2) contained information about the current visual input, about the granularity of the forthcoming categorical decision, as well as perceptual expectations about the upcoming visual stimulus. The middle temporal gyrus, the anterior temporal lobe, and the inferior frontal gyrus were also involved in the categorization process, constituting an attention and control network that modulates perceptual processing. These findings provide further evidence that early visual processes are driven by conceptual expectations and task demands.

## Introduction

Compared to most other mammalian orders, primates are predominantly visually oriented. This is supported by an extended cortical system, including the so-called dorsal and ventral cortical visual streams (Ungerleider and Mishkin 1982). It has been proposed that the ventral stream, specialized in object recognition, evolved to enable foraging of ephemerally ripe fruits (Dominy and Lucas 2001; Regan et al. 2001). Others have suggested that the ventral stream evolved to aid in face recognition, necessary for the high social demands of most primate societies (Boinski and Garber 2000). The ventral visual stream allows ever more abstract processing of stimulus features, including categorical abstractions: the ability to parse a continuous world into discontinuous categories, even when the sensory input varies continuously (Freedman et al. 2003). Indeed primates have an extraordinary capacity to make subtle categorical distinctions (e.g., rhesus macaques (Orlov et al. 2000); capuchin monkeys (Smith et al. 2012); and chimpanzees (Oden et al. 1988)).

Humans in particular have a remarkable capacity to arrange and rearrange perceptual input according to different categorizations. A Labrador can be a dog, a pet, a mammal, a companion, or a predator, dependent on the context. Classical theories on visual perception would hold that early visual processing is independent of these categorizations (Kandel and Wurtz 2000). Only in higher visual areas do these categorizations and connotations shape information processing. In line with this, it has been shown that task properties have an impact on visual processes primarily in higher visual areas (Bracci et al. 2017; Harel et al. 2014). However, recent work on visual processing places strong emphasis on the adaptive nature of neural coding, even in early visual cortex (Gilbert and Li 2013; Kok et al. 2012). Within this “active vision” framework, the type of information required to be extracted from a stimulus is expected to influence early visual processing. This notion is also compatible with recent data suggesting that incoming perceptual input is compared with top–down driven perceptual predictions, so-called “predictive processing” (Friston and Kiebel 2009; Rao and Ballard 1995). What is further propagated to other cortical sites is the so-called error signal, the difference between the actual and predicted input (Clark 2013).

In order to investigate the extent to which prior goals can influence early visual areas we scanned 25 healthy subjects during a simple semantic discrimination task. Subjects were asked to answer questions about an upcoming animal picture. The questions were on 2 different levels: basic-level questions (e.g., “Is this a frog?”) or superordinate-level questions (e.g., “Is this an amphibian?”). In line with active vision theories we found evidence of task modulation in multiple visual areas, including V1, peristriate areas, fusiform gyrus, middle temporal gyrus, anterior temporal lobe, and inferior frontal gyrus. In line with predictive processing theories, we found evidence of expectations in V1, V2, and V3: a question about an upcoming animal gave rise to anticipatory activation in these occipital regions that is specific for that animal. Together these results are in line with the idea that perception is an adaptive, task-dependent, and with the influence of predictive process already at the early visual cortices.

## Materials and Methods

### Participants

Twenty-five participants took part in a behavioral experiment, and 25 different participants took part in the imaging part of this study (based on previous decoding studies). Of the imaging participants, 6 were excluded in total: 1 due to technical issues, and 5 due to insufficient performance (less than 2 correct trials in any of the conditions per run). The remaining pool consisted of 19 participants (of which 12 were female) between 18 and 32 years of age (mean age 23.6 years, standard deviation 3.5 years). All participants had normal or corrected-to-normal vision and were right-handed according to the Edinburgh handedness assessment (Oldfield 1971). Participants had no history of neurological or psychiatric disorders, and gave written informed consent. All participants mastered the Dutch language at a native level, were recruited through the Radboud University online recruitment system and received €5,—for participation in the behavioral study (0.5 h) or €15,—for participation in the imaging study (1.5 h). The study was approved by the local ethics committee.

## Experimental Setup

### Behavioral

Participants sat in front of a computer screen at roughly 70 cm. Stimuli consisted of 3 target animals (a giraffe, a frog, and a dog), and were presented using PsychoPy version 1.83.03 (Peirce 2007). We chose a relatively low number of different stimuli (animals) to make the behavioral task comparable to the imaging task, in which only 2 different animals could be used. Five different pictures were used for each animal. The target animals were interspersed with nontarget animals (bat, chicken, cow, tiger, lizard, tuna, bird, monkey) to make the task more interesting. The questions for the nontarget animals were the same as for the target animals. All images—both target and nontarget—consisted of an animal against a white background. Images were matched for size and luminance.

Participants performed 5 blocks of 80 trials each. Each trial started with a question at 2 possible levels: the basic level or a superordinate level. An example of a basic-level questions is “Is this a frog?” and an example of a superordinate-level question is “Is this an amphibian?”. After the question that was presented for 2000 ms an animal picture was presented. The options “yes” and “no” were visible below the animal picture. The location of the 2 answer options was not randomized to facilitate fast responses. Participants had 2000 ms answering time, and answered by pressing 1 of 2 buttons on a button box (BITSIBOX) that was connected through a USB-port. There was a 4000 ms intertrial waiting time.

### Imaging

**Figure 1:**
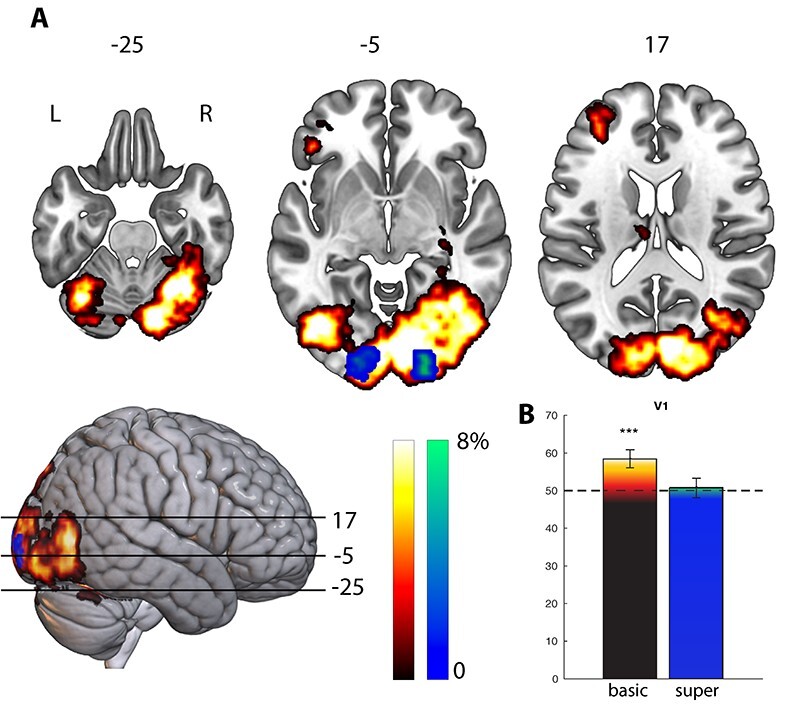
Overview of 1 trial.

For the imaging experiment, only 2 target animals were used (frog and dog) in order to maximize statistical power and keep scanning time below 1 h. Stimuli were again presented using PsychoPy version 1.83.03 (Peirce 2007), and projected onto a screen at the back of the scanner. The screen was visible to the participant through a mirror mounted on the MR head coil. The participants held 2 button boxes, 1 in each hand, and used their index fingers to press a button to perform the task. Each question was presented for 2000 ms, followed by a 4000 ms delay consisting of a gray screen. After this an image of an animal was presented against a white background. No answering options were presented at this stage, to prevent motor preparation. In order to avoid bias effects, the proportion of congruent to incongruent question–image pairs was kept at 0.5. Most animals were frogs and dogs (matched in size), interwoven with additional animals that were not used in subsequent analyses. Five different dog images and 5 frog images were used, to prevent low-level retinotopic features and test the concept “dog” and “frog”, rather than a specific picture. The animal pictures were presented for 1000 ms after which another gray screen was presented for 4000 ms After this the answer options were presented (“yes” and “no”), on each side of the screen, corresponding to a button in the left or right hand of the participant. The position of the 2 answer options was randomized in order to prevent anticipatory motor preparation. Due to this randomization and the 4000 ms delay between target stimulus and answer options—required to acquire enough scans for perform multivariate analyses of the imaging data—we were not able to analyze reaction times in the imaging part of this study. Immediately after answering the question there was a 1000 ms intertrial interval, after which the next trial began (see Fig. 1). Trial order was randomized. All trials that were not answered within 2000 ms were discarded. Since the intertrial interval began right after the participant pressed a button (not waiting for the 2000 ms maximum response time), a natural jitter occurred. Only congruent trials (trials for which the correct answer was “yes”, being 50% of the trials) were used for further analysis. Each participant performed 5 runs of 40 trials each.

### Image Acquisition

A 3 T Siemens Prisma (Erlangen, Germany) scanner with a 12-channel head coil was used to collect functional magnetic resonance imaging (fMRI) data. During the task T2^*^-weighted echo-planar images (EPI) were acquired, using a multiband multiecho sequence (time repetition [TR] = 1500 ms; time echo [TE_1]_ = 13.40 ms, TE_2_ = 34.42 ms, TE_3_ = 55.44 ms; flip angle = 75°). Each volume consisted of 84 × 84 × 64 isotropic 2.5 mm voxels. Additionally, a structural T1-weighted MPRAGE image was collected for anatomical localization (TR = 2640 ms; flip angle = 11°; 0.8 mm isotropic voxels), and a fieldmap scan (echo time: 4.7 ms; 60°: flip angle; 2.4 × 2.4 × 2 mm voxels).

### Data Analysis

The EPI images corresponding to the different echo times were aligned to the first echo of the first volume of each run. They were then combined using a weighted average and realigned to the first echo of the first scan of the run. These combined images were preprocessed using SPM12 (http://www.fil.ion.ucl.ac.uk/spm). A voxel displacement map (VDM) was calculated using the fieldmaps, and the images were unwarped using this VDM.

### Univariate Analysis

A general linear model (GLM) was estimated using only the regressors modeling effects on trials where the subject responded correctly. We included 4 regressors corresponding to the 4 conditions in the design (FROG BASIC; FROG SUPER; DOG BASIC; DOG SUPER), modeled as “box cars,” and convolved with a canonical hemodynamic response function. We also included 6 movement parameters as regressors of no interest. The contrasts FROG>DOG, DOG>FROG, BASIC>SUPER, and SUPER>BASIC were created in a GLM using SPM12 and MATLAB. The resulting contrast images were normalized to the MNI152 standard brain (third degree B-Spline interpolation) and smoothed with a 2 × 2 × 2 mm FWHM kernel. These images were used in a group-level analysis (1-sided *t*-test). None of the contrasts yielded a significant result (*P* > 0.001 voxel threshold, family-wise error corrected at the cluster level [FWE_C_]).

### Multivariate Decoding

A GLM was estimated using only the regressors modeling effects on trials where the subject responded correctly. The input images were not normalized or smoothed. We included 4 regressors corresponding to the 4 combinations of animals and question levels in the design (DOG BASIC, DOG SUPER, FROG BASIC, and FROG SUPER). Additionally, 4 regressors corresponding to the question presentation were included (Q-DOGBASIC, Q-DOGSUPER, Q-FROGBASIC, and Q-FROGSUPER). Regressors were modeled as a box-car at the time of the presentation of the animal picture (6000–8000 ms from trial onset), or the question presentation (0–2000 ms), convolved with a canonical hemodynamic response function. Again, we included 6 movement parameters as regressors of no interest as well. The condition-, voxel-, and run-wise parameter estimates of the resulting GLM were subsequently used as input for the multivariate analyses.

We employed a multivariate pattern analysis (MVPA) based on the GLM using The Decoding Toolbox (TDT; Hebart et al. 2015). For this, unsmoothed and non-normalized estimates were used. A searchlight classifier (12 mm radius) using libSVM (Chang and Lin 2011) was trained and tested in 3 different setups. These analyses were:

1. **Animals:** Dogs versus frogs at the basic level, and dogs versus frogs at the superordinate level
2. **Anticipation:** Dogs from frogs in a cross-modality (questions and images) cross-validated setup
3. **Levels:** Basic-level versus superordinate-level questions (irrespective of the animal)

For Analysis 1 (animals) a classifier was trained on 4 out of the 5 runs, and the remaining run was used to test the classifier’s performance. This was repeated 5 times, each time leaving out a different run (a leave-one-run-out procedure). For Analysis 2 (anticipation) the classifier was trained on the questions (“Is this a frog?” vs. “Is this a dog?”) and tested on the presented images (and vice versa). Finally, for Analysis 3 (levels) the contrast basic-level versus superordinate-level questions were classified using a leave-one-run-out procedure. This was done both irrespective of the stimulus identity (dog or frog).

All analyses resulted in decoding accuracy maps per subject. These maps were normalized to MNI space and used in a group-level analysis (1-sided *t*-test, *P* < 0.001 voxel threshold, FWE_C_).

For the generation of anatomy-based regions of interests (Analyses 1 and 2) we used the internal SPM maximum probability tissue atlas, which is in turn based the OASIS project (http://www.oasis-brains.org). Regions of interest (ROIs) were created in MNI space, and converted to individual brains using the inverse normalization matrix that was created during normalization step for group-level analyses. Activity-based ROIs were created using the SPM-based tool “Marsbar” (Brett et al. 2002).

**Figure 2:**
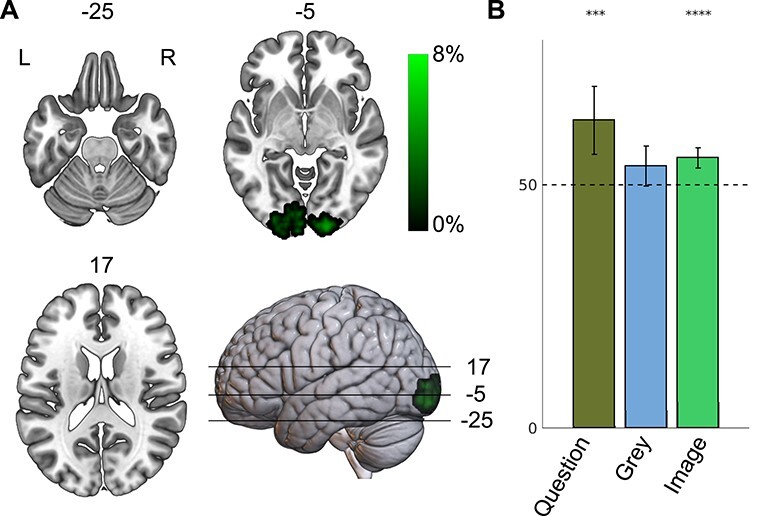
Panel (A): Decoding accuracy maps of above change decoding of frogs versus dogs in percentages (*P* < 0.001, family-wise error corrected at the cluster level) for basic-level decoding (red–yellow) and superordinate-level decoding (blue–green). Panel (B): ROI V1 comparison of decoding accuracy between basic-level (left) and superordinate level (right) decoding accuracy. Scale denotes percentage above-chance level (50%); maximum decoding values can be above this range. Whiskers show 95% confidence interval.

## Results

### Behavioral

Based on the classical work on object categorization (Rosch et al. 1976), we expected superordinate judgments to be more difficult, and hence slower, compared with basic-level judgments. We tested this in a behavioral experiment with a separate set of participants (*N* = 25). As expected, superordinate questions resulted in subtle, but significantly longer reaction times compared with basic-level questions (609 ms vs. 556 ms; *P* < 0.001; *t* = −3.7; df = 1; Cohen’s *d*: 0.48), suggesting that superordinate judgments are indeed more difficult than basic-level judgments. This is also reflected in accuracy score (95% for basic, and 87% for superordinate-level trials, *P* < 0.001; *t* = 4.6; Cohen’s *d*: 1.49).

### Univariate Results fMRI

Following the same reasoning that participants are less familiar with superordinate questions compared with basic-level questions, affecting retrieval of the relevant semantic items (as reflected in the behavioral results), we expected that superordinate questions would result in larger BOLD response in, among others, middle temporal gyrus and Broca’s complex. However, no univariate results were found.

### Multivariate Analysis 1: Dogs Versus Frogs

First, we wanted to test whether our classifier was able to dissociate between the basic types (i.e., animals) presented. Therefore, we classified dogs and frogs during image presentation following basic-level questions. For this we used a searchlight decoding analysis, which yielded information about the stimulus type (dog or frog) in the primary visual areas, extending anteriorly along the right fusiform gyrus, including the fusiform face area, and left anterior ventrolateral prefrontal cortex (pars orbitalis; see Fig. 2, panel A). Maximum decoding accuracy was 82%, and was located in left V1 (MNI −4, −90, 2, probability for V1 60%, according to the SPM Anatomy Toolbox (Eickhoff et al. 2005)). Note that we have used 5 different images per animal, differing in color, orientation, and size, so it is unlikely that the information in V1 represent retinotopic, color, or picture-specific information.

We then ran the same analysis, classifying frogs from dogs, this time during presentation of the same images following superordinate-level questions (e.g., “Is this a mammal?”). The decoding map obtained from this analysis was far less extensive, being restricted to left and right V1 and V2. Maximum decoding accuracy was 70% and located in left V1, bordering V2 (MNI −10, −102, 6; prob. V1 42%; prob. V2 24%). This suggests that the activation in V1 and V2 during perception of a stimulus is more variable when the stimulus follows a superordinate-level question compared with a basic-level question. In other words, even though the stimulus is the same, the type of question asked prior to its presentation affects signal processing in these early visual areas.

**Figure 3:**
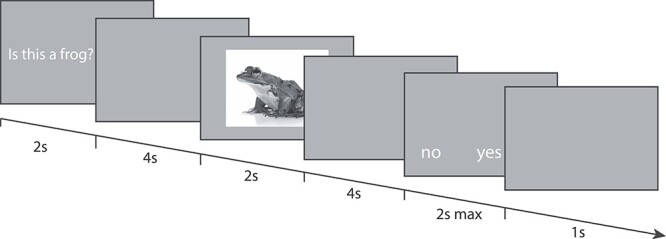
Results MVPA anticipation analysis. Panel (A): Decoding accuracy map above change (family-wise error corrected at the cluster level, *P* < 0.001) for cross-modal (questions and images) decoding of frogs and dogs. Panel (B): ROI comparison of the 3 types of cross-validation: questions–questions; questions–gray screen; and questions–images. Boxes portray decoding accuracy; whiskers signify a 95% confidence interval. Scale denotes percentage above-chance level (50%); maximum decoding values can be above this range.

To quantify the differences between stimulus presentation decoding following basic-level and superordinate-level questions, we performed an ROI analysis in V1 for the 2 conditions. Using the internal SPM brain atlas (which is based the OASIS project (http://www.oasis-brains.org) an ROI encompassing bilateral V1 was created and the average decoding accuracy of all voxels within this ROI was compared in the 2 conditions. A repeated measures ANOVA shows a significant effect for condition (*P* < 0.05). When all voxels within the anatomical region were averaged, above-chance classification was only present following basic-level questions. The searchlight analysis does show a significant V1 cluster for superordinate decoding, as can be seen in Figure 2, but this cluster is smaller and of a lower decoding accuracy than the basic-level cluster.

### Multivariate Analysis 2: Anticipation

The fact that V1 processes visual input after basic-level and superordinate-level questions differently suggests that the activation in this occipital area is not solely driven by the perceptual input, but a combination of the input and task properties. This would suggest that already at the moment of stimulus presentation V1 is prepared for the stimulus. In our experimental setup this means that the questions would have a priming effect on V1. In order to check this priming, we cross-decoded questions and images: we trained the classifier on classifying the 2 questions (“Is this a frog?” and “Is this a dog?”, i.e., the first 1000 ms of the trial), and tested the classifier on the time series corresponding to the frog and dog image presentation, and vice versa. This was done again using a searchlight approach. Only trials that were answered correctly were used. We found above-chance cross-decoding in bilateral V1, V2, and V3v. Maximum decoding accuracy was 62% and located in left V3v (MNI −24, −92, −10, prob. for V3v 49%; see Fig. 3, panel A). This means that the question about an upcoming animal generates a spatial pattern in early visual cortices that is comparable to the pattern that accompanies actual perception of the actual animal. Again, note that this cannot be due to specific low-level retinotopic patterns, as 5 different images (differing in size, color, orientation and perspective) per animal were used.

An alternative explanation of the finding of anticipatory activation in the occipital cortex could be in terms of temporal bleeding: activation patterns corresponding to word form during the question phase could still be present during the image presentation, and can therefore be picked up by the classifier. This alternative explanation entails that the gray screen between questions and images also contains the question information. In order to test this possibility, we selected the cluster with the highest decoding accuracy in the previous analysis, which was located in left V3v (MNI −24, −92, −10). In a spherical ROI (*r* = 5 mm) around the peak of this cluster we compared decoding accuracy of the questions themselves, and we cross-decoded image presentations and the last 2 s of the gray screen in between question and image presentation, both using a leave-one-run-out procedure. A repeated measures ANOVA showed a significant effect for condition (*P* < 0.05), with question–question decoding being significantly different from question–gray decoding. After correcting for multiple comparison (Bonferroni, 3 conditions in total), classifying questions resulted in a decoding accuracy significantly higher than chance (decoding accuracy 63%, *P* < 0.005), however, no decoding accuracy significantly higher than chance was found for the question–gray screen cross-validated test (decoding accuracy 54%, *P* = 0.04, α = 0.0167, see Fig. 2, panel B). The results of both the ANOVA and the separate *t*-tests can only mean that whatever pattern corresponding to the visual form of the question is still present in this cluster at the time of image presentation, it cannot account for the cross-validated decoding between questions and images, since it would have to be present during the gray screen as well. In all, the questions “Is this a dog?” and “Is this a frog?” prepare the visual system for the upcoming stimulus.

### Multivariate Analysis 3: Question Levels

**Figure 4:**
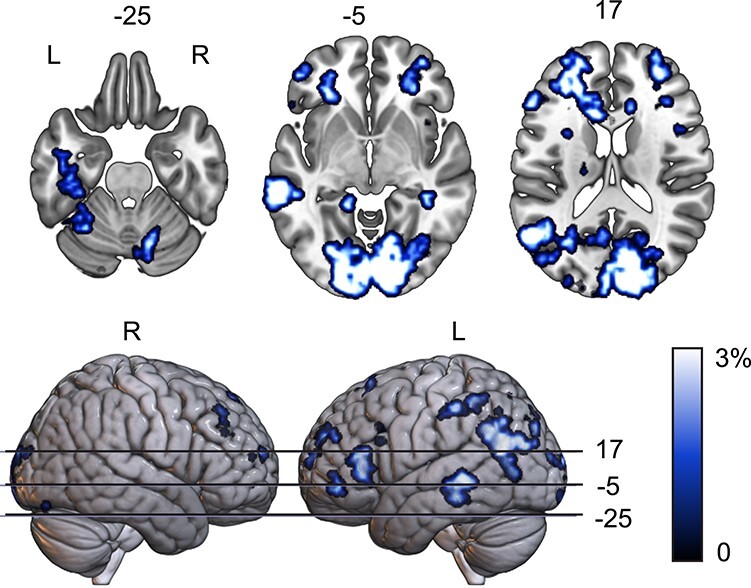
Decoding accuracy map above change (family-wise error corrected at the cluster level, *P* < 0.001) decoding of question levels (basic-level vs. superordinate level questions). Scale denotes percentage above-chance level (50%); maximum decoding values can be above this range.

Finally, we investigated what the impact of the question level would be on the way the images were processed. This would give us insight in whether the 2 levels impact the processing in early visual cortices, irrespective of the stimulus identity. For this we trained the classifier on basic-level question trials versus superordinate question trials (ignoring stimulus identity). Above-chance decoding (*P* < 0.001, FWE at the cluster level) was found in an extensive (mostly left-lateralized) cortical network (see Fig. 4), including the V1 and V2, left inferior frontal gyrus (including the pars opercularis (BA44) and the anterior part of the pars triangularis (BA45), left and right middle frontal gyrus, inferior orbitofrontal cortex. In the temporal cortex, stimulus level could be decoded from left middle temporal gyrus, ventral anterior lobe, and left temporoparietal junction.

Maximum decoding accuracy was in right V1 (decoding accuracy 71%; MNI: 8, −92, 6; prob. for V1: 61%). These findings show that 2 identical images are processed differently depending on the task, already as early as V1. Interestingly, these clusters overlap with the peak cluster from the stimulus anticipation decoding analysis above, suggesting that the anticipatory activation is task dependent as well.

## Discussion

We have shown that the nature of a stimulus (dogs or frogs) can be decoded from the fMRI data, primarily in left and right V1 and V2, and the right fusiform gyrus. Interestingly, the decoding accuracy was strongly dependent on the viewing task. Decoding image perception following superordinate-level questions was significantly less than following basic-level questions. This suggests that the activation in early visual areas is not solely driven by perceptual input, but a combination of the input and task properties, in line with “active vision” theories. This stronger decoding accuracy may be partly driven by the occurrence of more concrete predictions upon basic-level questions, but not entirely, since the cortical surface from which we can decode frogs and dogs is much larger than the cortical surface from which we can validate predictions.

Previous work shows that task properties (i.e., physical vs. semantic judgments) have an impact on the processing of object stimuli at several cortical sites, including ventral temporal and prefrontal regions (Harel et al. 2014). It has been shown that the usability of a presented object (e.g., tool vs. nontool) affects the occipitotemporal cortex differently (Bracci et al. 2017). Similarly, Nastase et al. (2017) found differences in brain response for a taxonomic versus an ethological judgment task in multiple brain regions, including occipital areas. These studies all found task-dependent processing of visual information, but only outside the primary visual areas. We, however, did find task dependence activation in primary visual areas. This may be due to the fact that our study has fewer categories (i.e., only 2 categories in 2 different tasks), compared to previous studies, which enhances statistical power drastically.

We speculated that prefrontal areas, specifically inferior frontal gyrus would be involved in modulating the activity in both temporal and visual areas. Indeed, these areas all seem to contain information about the task and stimulus identity, as reflected in above-chance decoding accuracy in Analyses 1 and 3.

The primate is an inherently visual animal, which is reflected in its elaborate visual system, including the so-called dorsal parietal and ventral temporal streams. It has been argued that the ventral, temporal stream evolved to allow an ever more abstract processing of the visual stimulus, which might provide the basis for our categorization behavior (Murray et al. 2019). In the ape and human lineages, this ability is more developed and possibly expanded to multisensory information (Bryant et al. 2019). As such, we expected that a network of prefrontal, temporal, and visual areas would underlie our capacity to use conceptual knowledge to process visual input. The anterior temporal cortex and the middle temporal gyrus, both bimodal association areas, are known to be involved in categorical decisions (Patterson et al. 2007). Indeed, it was possible to decode the level of abstraction of the required processing of the stimulus ventral anterior temporal cortex and middle temporal gyrus. The middle temporal gyrus result is particularly interesting, as it is close to the part of the temporal cortex that has most expanded and reorganized in the human, compared to the macaque, brain (Mars et al. 2018; Van Essen and Dierker 2007). The level of abstraction of the question itself could be decoded in a much larger set of cortical areas, including the inferior frontal cortex. Interestingly, these frontal and temporal areas are connected by specific sets of white matter fibers (i.e., the arcutate fasciculus and the inferior fronto-occipital fasciculus), some of which are particularly extended in the human lineage (Eichert et al. 2020). Our results suggest the involvement of these systems in tuning early visual processing for efficient task processing.

These results are in line with the framing of perception as a dynamic and task-driven process, tailored to the current needs of the cognitive system. Enactivist theories argue that cognition is not the representation of a pregiven world by a pregiven mind, but rather the enactment of a world and a mind on the basis of a history of the variety of actions that a being in the world performs. Within this view, perceptual capacities are embedded in a more encompassing biological, psychological, and cultural context (Varela et al. 1991). This active engagement with the environment is also suggested by more recent theoretical approaches to cognition (Hutto and Myin 2013; Myin and Degenaar 2014).

In line with this, we show that the early visual areas are tuned to those features in the environment that are relevant for the task at hand. The finding that left inferior frontal cortex shows significantly different activation patterns for basic-level and superordinate level judgment tasks suggests that the control this area exerts is not confined to behavioral control, but control over perceptual processing as well (Higo et al. 2011). This could also explain the absence of a univariate effect in our comparison of basic-level and superordinate-level trials. When perception is not a neutral process, but sense-making from the start, it would be equally task-driven in both conditions.

The finding of a behavioral difference suggests that the 2 decision processes (basic vs. superordinate) are not equally difficult. Superordinate categories are assumed to be less restricted in terms of visual input (e.g., the category “mammal” shows greater variance than the category “dog”). This increased difficulty is reflected by an increase in reaction time in the behavioral task. At the same time, the increased difficulty is reflected in a decrease of the cortical area from which the perceptual input could be decoded. Together with the fact that the increased difficulty is not reflected in gross brain activation (univariate BOLD result) during the viewing epoch of the imaging task, this suggests that the cortical areas are qually strongly but differently in nature involved in both tasks.

For efficient processing it is likely that task-dependent tuning to perceptual features primes the visual system before the actual perception. Indeed we have found evidence for expectations of upcoming stimuli in V1 and V2. A classifier trained on contrasting dog from frog questions was able to contrast dog from frog images as well. This anticipation surpasses low-level features such as lines and orientation, as different images were used per animal. This finding of modulation of V1 is in line with a recent reports showing that processes in V1 are biased by semantic categories (Ester et al. 2019) as well as action intentions (Gallivan et al. 2011). The finding of stimulus anticipation in V1 is in line with predictive coding accounts that recently have gained attention (Clark 2013; Rao and Ballard 1999). The influence of the level of the question we showed in V1 and V2 could partly be attributed to the presence of a concrete expectation of a dog or a frog in basic-level trials and the absence of such an expectation in superordinate trials, yet the cluster was far more extensive in the “levels” analysis compared with the anticipation analysis.

One could argue that the decreased decoding accuracy in superordinate trials is a consequence of differences in viewing behavior. Since participants were allowed to explore the presented image freely, it could be that viewing behavior in the superordinate condition was more variable. We did not collect eye-tracking data in order to quantify this potential difference, but the absence of a univariate results and the fact that the average difference in reaction time during the behavioral experiment between the 2 conditions was only 50 ms (note that the average saccades lasts 150–200 ms (Palmer 1999)), suggest that the contribution of differences in viewing behavior to the decoding effect is likely to be limited. Additionally, if indeed viewing behavior would play a role, one would expect this difference to be largest in the retinotopically organized occipital areas (e.g., V1). To the contrary, in our results, above-chance decoding is “preserved” in V1 and V2, and absent in more complex visual areas.

We cross-decoded questions and images, and questions and the gray screen between images and questions in order to check the nature of the anticipation present in early visual areas. The fact that we could not cross-validate questions and gray screens, but we could cross-validate questions and images suggests that the anticipation is a more complex phenomenon than mere sustained activity, and points toward more dynamical explanations (see for instance Wolff et al. 2017 for an example of such a model for working memory).

In all, these findings suggest that early visual areas are not processing visual input in a neutral or passive way. Rather their activation seems to be the result of anticipatory, task-driven processes, constituting an active engagement with the environment. These findings could have profound consequences for our understanding of how concepts are processed by the brain. Apparently, a frog-as-a-frog is processed differently than a frog-as-an-amphibian. Even the activity in the left temporal pole, which has been suggested to accommodate task-independent concept representations (Patterson et al. 2007), shows task-dependent modulation in our study. Our findings are thus more in line with classical pragmatists (Sellars 1963) and more recent enactivist (Hutto and Myin 2013) theories that suggests that the identity of a concept is (partly) grounded in the way a concept is used. This could provide a highly speculative, but interesting new explanation for the reported dependence of conceptual knowledge on perceptual systems (Barsalou et al. 2003): concepts can be seen as perceptual capacities, driven by parietal and prefrontal control processes, rather than internal representations. When concepts are much more use-based, as hypothesized, the question moves from how concepts are represented (Patterson et al. 2007), to how concepts acquire the stable character that they have in their (communicative) use. Part of the stability may be dependent on invariant structures outside of the brain, for instance in social practice or other behavioral patterns.

## Supplementary Material

Supplementary_material_bhab163
