# Supplementary material for: The Anticipatory and Task-Driven Nature of Visual Perception
Source: Cereb Cortex. 2021 Sep 7;31(12):5354–62. doi: 10.1093/cercor/bhab163 (PMC8567999; doi:10.1093/cercor/bhab163)
Supplement: Supplementary_material_bhab163 [file supplementary_material_bhab163.docx]

# Supplementary material

**On the issue of temporal bleeding**

In order to verify the nature of the cross-validated (questions and images, multivariate analysis 2) decoding map, we have used a small ROI around the decoding peak, which was located in right V2, and cross-validated questions with time series corresponding to the grey screen in between questions and images. Yet, the reader may wonder what the decoding map would look like if we do not restrict this analysis to such a small ROI. To accommodate this reader, we have performed this analysis. We found some small signs of ‘temporal bleeding’ in V1 and V2, barely passing significance. No other cortical or subcortical sites were found. For comparison, we have added our findings of cross-decoding questions and images (on the right). Comparing the two decoding maps clearly shows that even though there maybe a few voxels showing temporal bleeding effects in occipital cortex, they cannot account for the finding we report in the paper.


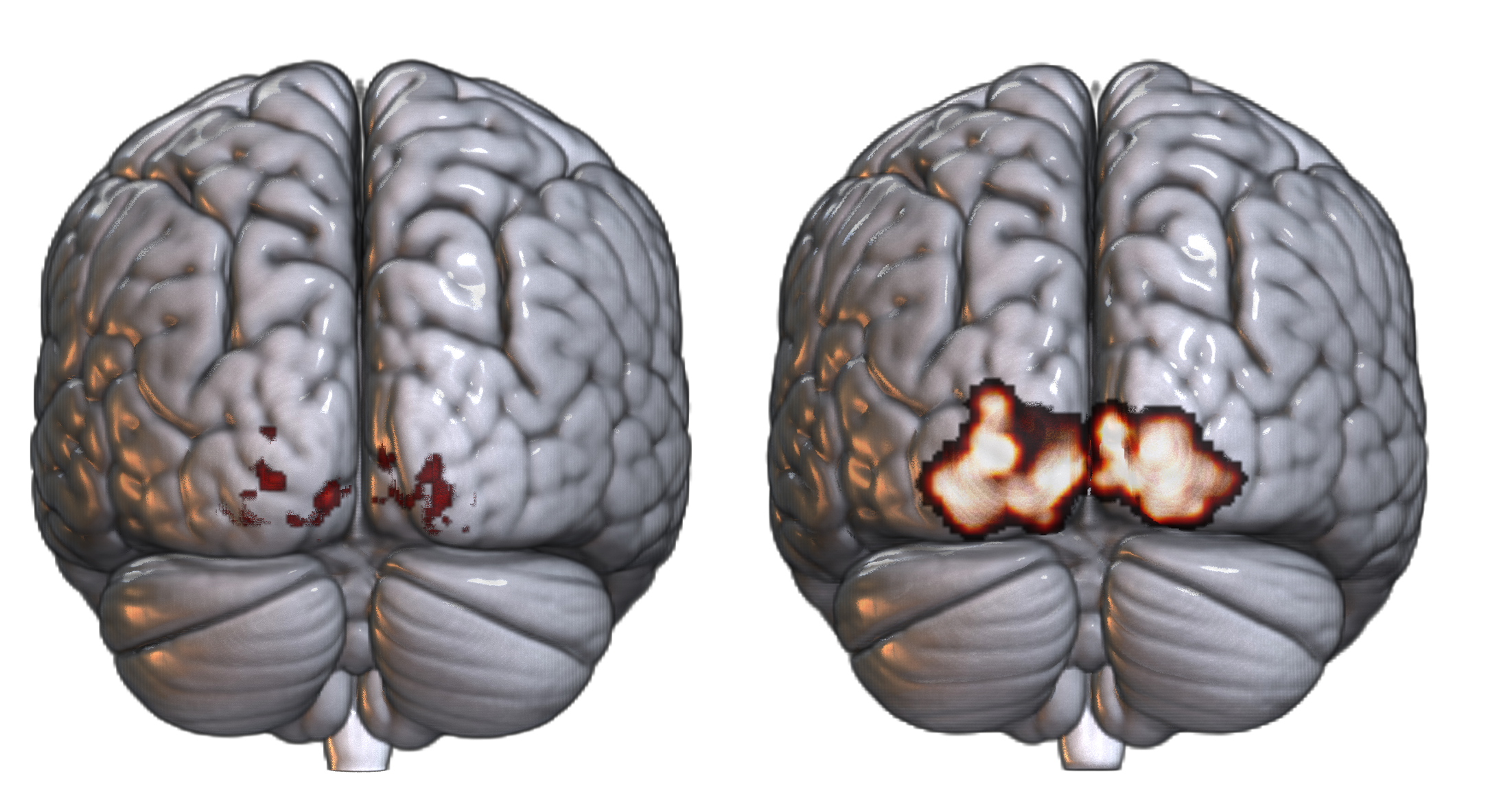


We chose not to report this extra analysis in the main paper for two reasons. First it would interrupt the flow of the paper too much, because in essence it is an extra check of an extra check. Second, to do a whole-brain searchlight decoding analysis, without a clear and a priori research question we fear of asking too much from our data, which would render the found decoding results hard to assess.

## Tables with Classification scores

**Analysis 1: Dogs vs Frogs Basic Level**

| **Region** | **Cluster Size** | **x** | **y** | **z** | **p(FWE-corr)** |
| --- | --- | --- | --- | --- | --- |
| Occipital Cortex | 11938 | 22 | -60 | -24 | 0 |
|  |  | 40 | -60 | -10 |  |
|  |  | 12 | -82 | 20 |  |
| left Thalamus | 43 | -8 | -22 | 10 | 0,023 |
|  |  | -12 | -14 | 14 |  |
| left aPFC | 90 | -34 | 54 | 22 | 0 |
|  |  | -32 | 44 | 16 |  |
|  |  | -30 | 36 | 18 |  |
| left V2 | 41 | -6 | -64 | -14 | 0,03 |
|  |  | 4 | -62 | -12 |  |
| left dlPFC | 51 | -38 | 44 | 0 | 0,009 |
|  |  | -44 | 30 | -4 |  |
|  |  | -40 | 34 | 2 |  |

**Analysis 1: Dogs vs. Frogs Super Level**

| **Region** | **Cluster Size** | **x** | **y** | **z** | **p(FWE-corr)** |
| --- | --- | --- | --- | --- | --- |
| Left V2 | 152 | -12 | -100 | 4 | 0 |
|  |  | -18 | -90 | -6 |  |
|  |  | -12 | -90 | 0 |  |
| Right V2 | 72 | 20 | -94 | -4 | 0,001 |
|  |  | 22 | -98 | 4 |  |

**Analysis 2: Anticipation**

| **Region** | **Cluster Size** | **x** | **y** | **z** | **p(FWE-corr)** |
| --- | --- | --- | --- | --- | --- |
| Left V2 | 341 | -24 | -88 | -10 | 0 |
|  |  | -6 | -100 | -6 |  |
|  |  | -4 | -86 | -4 |  |
| Right V2 | 218 | 16 | -92 | -12 | 0 |
|  |  | 18 | -96 | -4 |  |
|  |  | 8 | -92 | -2 |  |

Analysis 3: Levels

| **Region** | **Cluster Size** | **x** | **y** | **z** | **p(FWE-corr)** |
| --- | --- | --- | --- | --- | --- |
| left V2 | 727 | -18 | -62 | 48 | 0.000 |
|  |  | -40 | -72 | 20 |  |
|  |  | -36 | -64 | 50 |  |
| Right V2 | 3108 | 6 | -76 | 16 | 0.000 |
|  |  | 18 | -90 | 2 |  |
|  |  | -22 | -74 | 10 |  |
| Right Operculum | 149 | 48 | 18 | 16 | 0.000 |
|  |  | 36 | 44 | 4 |  |
|  |  | 54 | 24 | 14 |  |
| left Middle Temporal Gyrus | 468 | -50 | -32 | -8 | 0.000 |
|  |  | -40 | -46 | 0 |  |
|  |  | -46 | -54 | 12 |  |
| - | 160 | -18 | -4 | 30 | 0.000 |
|  |  | -12 | -8 | 34 |  |
|  |  | -12 | -6 | 42 |  |
| left aPFC | 360 | -40 | 48 | 14 | 0.000 |
|  |  | -56 | 26 | 12 |  |
|  |  | -44 | 38 | 14 |  |
| left supramarginal gyrus | 75 | -52 | -40 | 42 | 0.000 |
|  |  | -60 | -42 | 40 |  |
|  |  | -48 | -44 | 50 |  |
| - | 114 | 16 | -56 | 38 | 0.000 |
|  |  | 26 | -56 | 34 |  |
|  |  | 18 | -62 | 44 |  |
| - | 63 | 36 | -42 | 28 | 0.001 |
|  |  | 28 | -38 | 34 |  |
|  |  | 30 | -44 | 20 |  |
| right Angular Gyrus | 40 | 50 | -48 | 36 | 0.019 |
|  |  | 50 | -42 | 28 |  |
| left Frontal Eye Fields | 80 | -36 | 22 | 38 | 0.000 |
|  |  | -24 | 22 | 32 |  |
|  |  | -22 | 16 | 38 |  |
| Cerebellum | 108 | -18 | -62 | -22 | 0.000 |
|  |  | -22 | -68 | -16 |  |
|  |  | -24 | -60 | -32 |  |
| Right dlPFC | 44 | 30 | 30 | 32 | 0.011 |
|  |  | 28 | 40 | 36 |  |
|  |  | 20 | 32 | 32 |  |
| - | 48 | -4 | -20 | -12 | 0.006 |
|  |  | -12 | -20 | -10 |  |
| Left Pars Orbitalis | 45 | -28 | 32 | -10 | 0.009 |
| Left Premotor Area | 48 | -34 | 4 | 40 | 0.006 |
|  |  | -32 | 4 | 48 |  |
|  |  |  |  |  |  |
